# Multi-omics profiling reveals associations between gut microbiota and olfactory gene expression in mosquitoes

**DOI:** 10.3389/fcimb.2025.1745848

**Published:** 2026-01-26

**Authors:** HeTing Gao, JianHang Li, LiFang Liu, ZhenYu Gu, HaoTian Yu, Dan Xing, Teng Zhao, ChunXiao Li

**Affiliations:** 1 State Key Laboratory of Pathogen and Biosecurity, Beijing Institute of Microbiology and Epidemiology, Beijing, China; 2 Institute of Tropical Medical, Guangzhou University of Chinese Medicine, Guangzhou, China

**Keywords:** culex, gut microbiota, multi-omics association, olfactory, RNA-seq, vector biology

## Abstract

**Introduction:**

The interplay between gut microbiota and host physiological processes has been extensively studied in vertebrates, where it plays a crucial role in regulating appetite, emotion, immunity, and other physiological functions. However, whether a similar regulatory mechanism exists in insects remains unclear, especially regarding the long-distance regulation of olfactory function. This study focused on three *Culex* subspecies (*Culex quinquefasciatus*, *Culex pipiens pallens*, and *Culex pipiens molestus*) that are closely related but exhibit significant differences in olfaction-dependent ecological habits. By integrating antennal transcriptomic and gut metagenomic data, we systematically analyzed the expression characteristics of olfactory-related genes, the structure of gut microbial communities, and their intrinsic associations.

**Methods:**

We integrated antennal transcriptomic and gut metagenomic sequencing to analyze olfactory-related gene expression, gut microbial community structure, and their intrinsic associations in male and female individuals of the three *Culex* subspecies. Bioinformatics analyses included differential gene screening, functional enrichment, microbial taxonomic annotation, and Spearman correlation analysis.

**Result:**

The results showed that a large number of sex-specific and species-specific differentially expressed genes (DEGs) were identified in the antennae of the three *Culex* subspecies. Among these, 345 DEGs were shared sex-specific genes across species, which were significantly enriched in pathways such as odor binding, signal transduction, and xenobiotic metabolism. At the phylum level, the gut microbial composition was dominated by Proteobacteria, Bacteroidetes, and Firmicutes, showing a conserved structure; at the genus level, 11 dominant genera (including *Wolbachia*, *Elizabethkingia*, and *Asaia*) exhibited distinct species-specific distribution patterns. Diversity analysis revealed that the gut microbial richness of male individuals was significantly higher than that of females, and the β-diversity showed an obvious "sex clustering" pattern.Correlation analysis further indicated that 152 DEGs were significantly correlated with 107 microbial genera. Among them, olfactory-related genes were closely associated with several core genera (e.g., *Wolbachia*, *Asaia*, *Serratia*). Gut microbes may remotely regulate the expression and function of olfactory genes in antennae through metabolites or signaling molecules, thereby influencing mosquito behaviors such as host localization, mating, and oviposition.

**Discussion:**

This study reveal the intrinsic association between gut microbes and olfactory function in *Culex* mosquitoes, providing a new perspective for understanding the "microbe-host" cross-organ regulatory mechanism and laying a theoretical foundation for the development of novel mosquito vector control strategies based on microbial or olfactory interference.

## Introduction

In vertebrates, the functional connections between gut microbiota and the central nervous system form a critical regulatory pathway, which has been confirmed to be extensively involved in various physiological processes of the host, including appetite regulation, emotional perception, and immune defense (Desai et al., 2016; Kim et al., 2022; Carbia et al., 2023; Siopi et al., 2023; Yu et al., 2024). Its core mechanism lies in the cross-organ communication between gut microbes and the central nervous system via metabolites or immune signaling molecules. For example, γ-aminobutyric acid (GABA) produced by gut microbiota metabolism can act on brain neurons through the bloodstream, influencing the host’s anxiety-like behaviors (Bravo et al., 2011; Strandwitz et al., 2019). Conversely, stress hormones secreted by the brain can also regulate the function of the intestinal mucosal barrier and the composition of gut microbiota in a reverse manner (Rykalo et al., 2024; Khan et al., 2025). The identification of functional connections between gut microbiota and the central nervous system has provided an important theoretical framework for analyzing “microbe-host physiology” interactions, advancing research in fields such as metabolic diseases and neurodegenerative diseases.

In insects, functional connections between gut microbiota and host physiology—analogous to the associations characterized in vertebrate gut-brain research—have been increasingly recognized. Gut microbes not only synthesize essential nutrients for the host and sustain basic physiological metabolism but also may be linked to insect nervous system function and behavioral phenotypes through metabolite signaling or signaling molecule interactions (Das De et al., 2022; Zeng et al., 2024; Zhong et al., 2024). Existing studies have shown that in Drosophila, acetic acid produced by intestinal lactic acid bacteria can activate the insulin signaling pathway in the central nervous system, which is associated with the regulation of host foraging behavior (Li et al., 2018; Kim et al., 2021; Chen et al., 2024). In honeybees, gut microbiota metabolites may modulate dance communication and homing ability by affecting brain neurotransmitter levels (Zhang et al., 2022a, b). However, research on the potential associations between gut microbes and olfactory function in mosquitoes remains scarce—despite the olfactory system being a core reliance for mosquito survival and pathogen transmission.

For mosquitoes, the olfactory system serves as the “navigational center for life activities,” as almost all key behaviors depend on the accurate recognition of olfactory signals: Female mosquitoes need to capture volatile substances released by hosts through olfaction to accurately locate hosts and complete blood-sucking, a process that is not only a necessary condition for ovarian development in most female mosquitoes but also a core link in their pathogen transmission (De Obaldia et al., 2022; Herre et al., 2022; Baik et al., 2024). Male mosquitoes, on the other hand, rely on olfaction to detect sex pheromones released by females, ensuring the smooth completion of mating behavior (Barredo and DeGennaro, 2020; Bansal and Sen, 2025). Meanwhile, mosquitoes also use olfaction to identify metabolic odors of specific microbes in water-accumulating environments, selecting suitable oviposition sites to ensure the survival of their offspring (Afify and Galizia, 2015; Zhao S. et al., 2024). It can be said that the performance of olfactory function directly determines mosquitoes’ reproductive success rate, population continuity, and pathogen transmission risk, making it a key biological trait for mosquitoes to adapt to the ecological environment.

*Culex quinquefasciatus* (*Cx. quinquefasciatus*), *Culex pipiens pallens* (*Cx. p. pallens*), and *Culex pipiens molestus* (*Cx. p. molestus*) all belong to the *Culex pipiens* complex (Turell, 2012; Alout et al., 2025). As widespread urban sanitary pests globally, they are closely related with minimal genetic differences yet exhibit significant differentiation in olfaction-dependent ecological habits: *Cx. p. pallens* has a preference for human odors and is often active in human-inhabited areas (Ikeshoji, 1966); *Cx. quinquefasciatus* has a broader host range and can switch flexibly between humans and other animals (Gao et al., 2022); *Cx. p. molestus*, due to its autogeny (non-hematophagous egg maturation and oviposition), has lower dependence on host odors and tends to select special environments such as underground stagnant water for oviposition (Gu et al., 2022). This difference in olfaction-based ecological habits makes them ideal models for studying the association between gut microbes and olfactory function. Questions such as whether the difference in host odor preference among different subspecies is related to the differential expression of olfactory genes in antennae, and whether metabolites produced by gut microbes can affect olfactory gene expression or neural signal transmission through remain to be explored in relevant studies to propose potential regulatory mechanisms of mosquito olfaction.

This study takes *Cx. quinquefasciatus*, *Cx. p. pallens*, and *Cx. p. molestus* as research objects, integrating antennal transcriptome sequencing and gut metagenomic sequencing technologies to focus on analyzing the expression characteristics of olfactory-related genes in the antennae of the three mosquito species, the core composition of gut microbial communities, and the association network between the two. The study aims to clarify the interaction mechanism between gut microbes and mosquito olfactory function and providing theoretical support for the development of novel mosquito control strategies targeting the olfactory system or gut microbes.

## Materials and methods

### Experimental materials and sample collection

The *Cx. quinquefasciatus*, *Cx. p. pallens*, and *Cx. p. molestus* used in this study were all long-term domesticated strains in the laboratory. These strains were continuously reared for multiple generations under standardized conditions to ensure genetic stability and consistency in biological characteristics, thereby avoiding interference from wild population variations on the experimental results.

The environmental conditions for larval mosquito rearing were set as follows: the temperature was controlled at 26 ± 1°C, the relative humidity was maintained at 75% ± 5%, and the photoperiod was 14 hours of light/10 hours of dark (14L:10D). During the larval stage, commercial fish feed was used for regular feeding. After larval eclosion, the adult mosquitoes were still reared under the same environmental conditions as the larval stage. Adult mosquitoes were fed with a 10% sucrose solution to meet their basic nutritional needs. At 3–5 days post-eclosion, male mosquitoes and non-blood-fed female mosquitoes were collected for subsequent experiments—at this stage, the olfactory system of adult mosquitoes is fully mature, and the intestinal microbial community is stable.

Forty mosquitoes of each species were randomly selected for tissue separation, and all operations were performed under aseptic conditions to avoid external microbial contamination. First, the mosquitoes were anesthetized on ice, and then their antennae and intestinal tissues were carefully separated using sterile dissecting needles and forceps.

For antenna samples: Antennae from every 10 mosquitoes of the same species and gender were pooled into one biological replicate, and 4 biological replicates were set for each species-gender combination. For intestinal samples: the same pooling strategy as that for antenna samples was adopted—intestines from every 10 mosquitoes of the same species and gender were combined into one biological replicate, with 4 biological replicates also set for each species-gender combination.

After sample collection, all pooled antenna and intestinal samples were immediately frozen in liquid nitrogen to minimize RNA degradation and maintain the stability of the intestinal microbial community. subsequently, the frozen samples were transferred to a -80°C ultra-low temperature refrigerator for long-term storage.

### Nucleic acid extraction and high-throughput sequencing

Total RNA was extracted from antenna samples using the TRIzol method. After extraction, the integrity of RNA was detected by agarose gel electrophoresis, and the purity of RNA was determined using a NanoDrop nucleic acid detector. For total RNA with qualified purity and integrity, mRNA was enriched using magnetic beads conjugated with Oligo (dT). The enriched mRNA was fragmented and then used as a template to synthesize cDNA for the construction of strand-specific cDNA libraries. After passing the library quality inspection, 150 bp paired-end (PE) sequencing was performed on the Illumina NovaSeq 6000 sequencing platform to obtain raw transcriptome sequencing data of the antennae.

Total DNA was extracted from intestinal tissues using the QIAGEN 69506 DNeasy Blood & Tissue Kit. After total DNA extraction, the integrity of DNA fragments was detected by agarose gel electrophoresis, and the purity of DNA was measured using a NanoDrop. The qualified total DNA was fragmented, followed by end repair, adapter ligation, and PCR amplification steps to construct metagenomic sequencing libraries. After the libraries passed quality verification, high-throughput sequencing was conducted to obtain raw metagenomic sequencing data of intestinal microorganisms.

### Bioinformatics analysis

Raw transcriptome sequencing data were first subjected to quality assessment using Fastp (Chen et al., 2018) and Fastqc (de Sena Brandine and Smith, 2019) software, and low-quality reads were filtered out to obtain high-quality clean data. The clean data were aligned to the reference genome using Hisat2 (Kim et al., 2019) software. Based on the alignment results, transcript assembly was performed using Stringtie (Pertea et al., 2015) software, and quantitative analysis of gene expression levels was conducted for the assembled transcripts to obtain the gene expression matrix of each sample. The R package DESeq2 (Love et al., 2014) was used for screening differentially expressed genes (DEGs), with the screening criteria set as |log_2_FoldChange| > 1 and padj < 0.05, to obtain the set of differentially expressed genes. To clarify the pathways in which the differentially expressed genes exert their functions, the R package clusterProfiler was used to perform Kyoto Encyclopedia of Genes and Genomes (KEGG) (Kanehisa and Goto, 2000) pathway enrichment analysis and Gene Ontology (GO) enrichment analysis on the differentially expressed genes.

Raw metagenomic sequencing data were also subjected to quality assessment and filtering using Fastp and Fastqc software to remove low-quality reads and adapter sequences, resulting in clean data. To eliminate the interference of host genome, Bowtie2 (Langmead and Salzberg, 2012) software was used to align the clean data with the host genome sequence of the corresponding mosquito species and reads aligned to the host genome were filtered out to retain reads derived from microorganisms. Kraken2 (Wood et al., 2019) software was used for taxonomic annotation of microbe-derived reads, and Bracken software was used to correct the annotation results to realize quantitative analysis of the relative abundance of microbial species, thus obtaining the microbial species abundance matrix of each sample. In addition, Megahit software was used to assemble the microbe-derived reads into microbial genome drafts, providing a basis for subsequent functional gene annotation and metabolic pathway analysis.

The reference genomes were obtained by downloading the corresponding species’ genome data from the National Center for Biotechnology Information (NCBI) (Benson et al., 2013; O’Leary et al., 2016), with the following accessions: *Cx. quinquefasciatus*: GCF_015732765.1 (Behura et al., 2011), *Cx. p. pallens*: GCF_016801865.2 (Peng et al., 2021) and *Cx. p. molestus*: GCA_024516115.1 (Liu et al., 2023).

### Correlation analysis

The gene expression matrix and the microbial genus-level relative abundance matrix were preprocessed. The R package corrplot was used to calculate the Spearman correlation coefficient between differentially abundant microbial genera and the expression levels of differentially expressed genes. Due to the large number of gene-microbe pairs involved in a single analysis (multiple comparisons), false discovery rate correction (FDR correction) was required to control false positive results. The R package vegan was used to perform the Mantel test, and the Spearman correlation coefficient was used to measure the strength of the association between the two distance matrices, which could reveal whether the overall changes in the intestinal microbial community were associated with the overall fluctuations in the antenna gene expression profile. β-diversity analysis is a core analytical method in microbial community ecology and biodiversity research, whose primary purpose is to quantify the degree of differences in community composition among different sample groups. Mantel test is a non-parametric statistical method, whose core purpose is to test the correlation between two matrices, and is particularly suitable for analyzing the association between community composition data and molecular data.

### Statistical analysis

All statistical analyses were conducted in the R 4.3.1 environment. For comparisons of gene expression levels and microbial abundance between groups, parametric or non-parametric tests were selected based on the distribution characteristics of the data. For Spearman’s rank correlation analysis (used to screen for potential associations between gut microbiota and mosquito genes), the screening thresholds were set as an absolute Spearman’s correlation coefficient (|R|) > 0.6 and a false discovery rate (FDR)-adjusted p-value (padj) < 0.05. The FDR correction was performed using the Benjamini-Hochberg method (implemented via the p.adjust() function in the base R stats package) to control for the false positive rate associated with multiple comparisons. The significance level for other statistical analyses was set to P < 0.05. All graphs were generated using the ggplot2 package in R.

## Results

### Quality control and basic statistics of high-throughput sequencing data

After filtering low-quality reads (Qphred < 20) and removing adapter sequences from the antenna transcriptome sequencing data, the sequencing data volume of 24 samples (3 *Cx.* subspecies × 2 sexes × 4 biological replicates) showed stable performance. The number of clean reads ranged from 2.90 Gbp to 4.59 Gbp, with an average of 3.62 Gb of clean data obtained per sample. After aligning the clean reads to the reference genome of *Cx. quinquefasciatus*, a total of 17,159 transcripts were successfully annotated, with an annotation rate of 92.96%, indicating a high coverage of the genome by the sequencing data. Base quality assessment results showed that the base quality is all above Q30, demonstrating that the sequencing data quality was reliable and sufficient for subsequent transcriptome analysis and multi-omics association analysis (**Supplementary File 1**, **Supplementary Table S1**).

After the same quality control process was applied to the gut metagenome sequencing data, the number of clean reads for 24 samples ranged from 7.21 Gb to 9.03 Gb, with an average of 8.12 Gb of clean data per sample. After removing the host genome sequences, the proportion of microbe-derived reads was between 82.35% and 88.67%, which effectively retained the sequencing information of gut microbes and provided high-quality data support for subsequent analysis of microbial community composition and diversity (**Supplementary File 2**, **Supplementary Table S2**).

### Transcriptome sequencing analysis of antennae from mosquito

**Figure 1:**
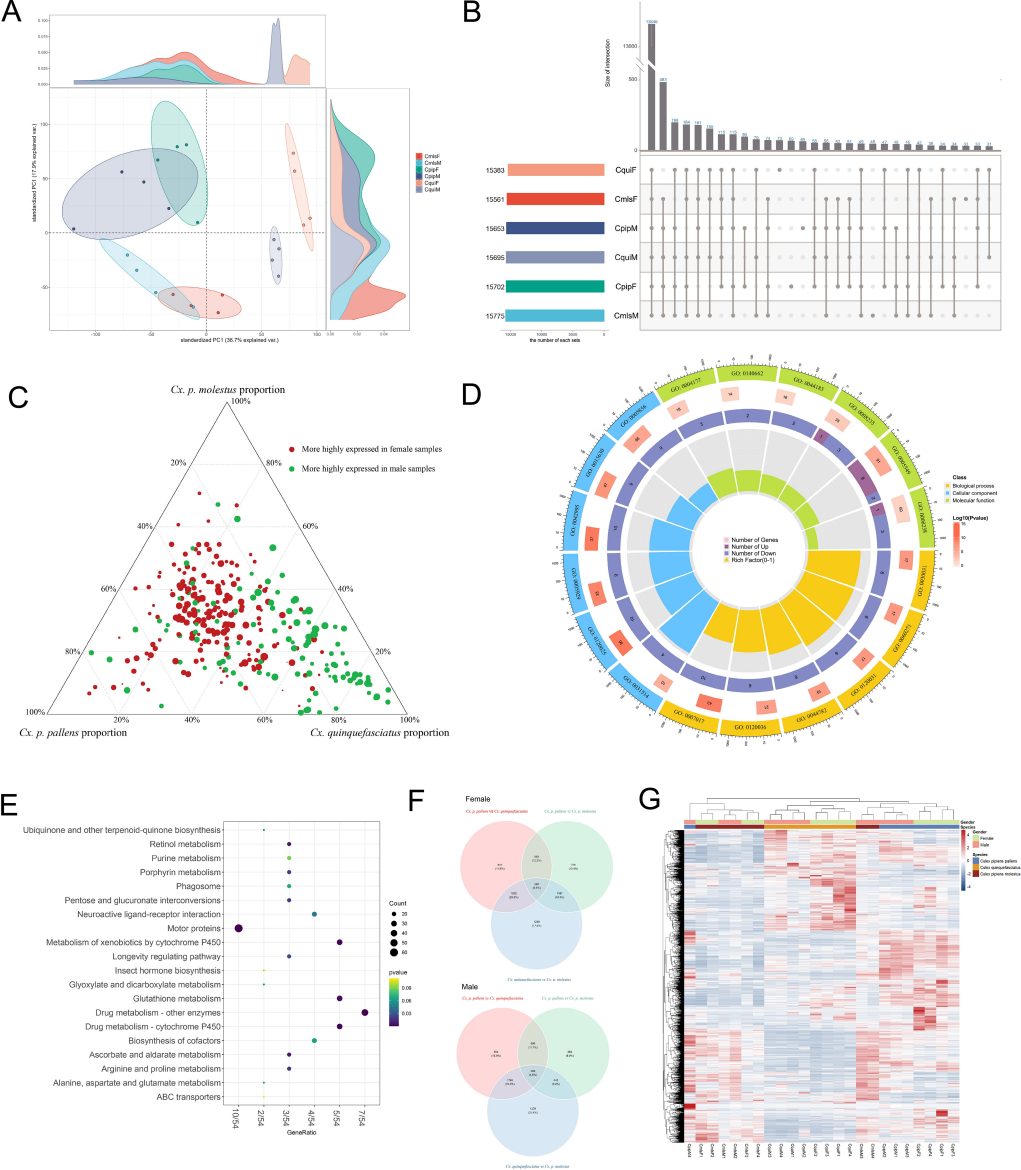
Transcriptome sequencing analysis results of mosquitoes. **(A)** PCA of the antennal RNA-Seq data. **(B)** Upset diagram for six groups transcrips. **(C)** Ternaryplot showing the distribution of sex-specific genes across three mosquitoes. **(D)** GO enrichment analysis of sex specific DEGs. **(E)** KEGG enrichment analysis of sex specific DEGs. **(F)** Venn diagram of species-specific differentially expressed genes. **(G)** Heatmap of overall expression pattern for inter-species DEGs.

Based on the 17,159 annotated transcripts, principal component analysis (PCA) was first performed to analyze the overall inter-group differences in gene expression. The results showed that the first principal component (PC1) and the second principal component (PC2) together explained 54.6% of the gene expression variation (PC1 contribution rate: 36.7%, PC2 contribution rate: 17.9%). From the perspective of group clustering characteristics, male and female samples of the same mosquito showed a significant aggregation trend, forming independent sub-clusters respectively, and the replicate samples within each group had high aggregation degree, indicating consistency in the effect of sex difference within species on gene expression. In contrast, samples of different mosquito with the same sex did not show an aggregation trend. it should be noted that, based solely on the gene expression variation explained by PC1 and PC2, we cannot elaborate on the differences in antenna transcriptome caused by sex factors (**Figure 1A**). Meanwhile, the universality of gene expression across different groups was counted, and the results showed that approximately 80% of the annotated genes (13,646/17,159) were expressed in all six groups (**Figure 1B**). This indicated that these genes might be conserved genes maintaining the basic physiological functions of *Culex* antennae, while the remaining genes showed group-specific expression, providing target objects for subsequent differential analysis.

#### Analysis of gene expression characteristics based on sex differences

The screening results of DEGs (|log_2_FoldChange|>1, padj<0.05) showed that there were significant sex-specific expressed genes in all three *Cx.* subspecies: the number of DEGs between males and females was the highest in *Cx. quinquefasciatus* (1,731), followed by *Cx. p. molestus* (1,622), and the lowest in *Cx. p. pallens* (1,376). Further analysis of the intersection characteristics of sex-related DEGs among the three *Cx* subspecies revealed that 345 genes showed differential expression between males and females in all three species, and the differential expression trends of these 345 common DEGs were completely consistent across the three *Cx.* subspecies: among them, 112 genes were highly expressed in females compared to males of the three species, and 233 genes were highly expressed in males compared to females. This suggested that these genes might play a core role in maintaining sex-specific behaviors of *Culex* mosquitoes (**Figure 1C**).

GO and KEGG functional enrichment analyses were performed on the 345 common DEGs. The GO enrichment results showed that: at the biological process level, the DEGs were significantly enriched in microtubule-related processes and cell structure assembly-related pathways, which are responsible for constructing cell structures, supporting cell morphology, and driving cell movement, and might be related to the development of the formation of signal transduction structures. at the cellular component level, they were enriched in functional pathways such as cytoskeleton and supramolecular complexes, which are associated with cell proliferation and population regulation. at the molecular function level, they covered multiple key functional pathways, including odorant binding, hydrolase activity, and transmembrane transporter activity (**Figure 1D**, **Supplementary File 3**, **Supplementary Table S3**).

KEGG pathway enrichment analysis showed that the 345 common DEGs were significantly enriched in 21 pathways (pvalue<0.05), including Drug metabolism, Metabolism of xenobiotics, Glutathione metabolism, Longevity regulating pathway, and Insect hormone biosynthesis, covering multiple dimensions such as drug metabolism, signal transduction, longevity regulation, and energy metabolism (**Figure 1E**).

#### Analysis of gene expression characteristics based on species differences

The screening results of inter-species DEGs (|log_2_FoldChange|>1, padj<0.05) divided by sex showed that the inter-species gene expression differences between females and males exhibited obvious sex specificity: among females, the number of DEGs was the highest between *Cx. quinquefasciatus* and *Cx. p. pallens* (4,681), followed by that between *Cx. p. pallens* and *Cx. quinquefasciatus* (3,989), and the lowest between *Cx. p. pallens* and *Cx. p. molestus* (3,159). among males, the number of DEGs was still the highest between *Cx. quinquefasciatus* and *Cx. p. pallens* (3,843), followed by that between *Cx. p. pallens* and *Cx. quinquefasciatus* (3,546), and the lowest between *Cx. p. pallens* and *Cx. p. molestus* (1,955) (**Figure 1F**).

To intuitively present the overall expression pattern of inter-species DEGs, a heatmap was used to show the expression profile of all inter-species DEGs. The heatmap clustering results showed that samples presented a relatively clear clustering trend according to the “species-sex” grouping: most male and female samples of the same species first clustered into one branch and then were distinguished from samples of other species. Moreover, the clustering distance between samples of *Cx. p. pallens* and *Cx. p. molestus* was closer, which was consistent with their biological characteristic of being more closely related. From the perspective of gene expression trends, three core expression modules could be divided into the heatmap: Module 1 contained 1,167 genes, which were specifically highly expressed in *Cx. quinquefasciatus* (both male and female samples) and significantly lowly expressed in *Cx. p. pallens* and *Cx. p. molestus*. Module 2 contained 1,148 genes, which were only highly expressed in *Cx. p. pallens*, showing strict species specificity. Module 3 contained 1,345 genes, which were highly expressed in both *Cx. p. pallens* and *Cx. p. molestus*, while their expression was inhibited in *Cx. quinquefasciatus* (**Figure 1G**).

GO functional enrichment analysis was performed on each group of inter-species DEGs separately, and the results showed that a total of 172 related pathways were enriched (padj<0.05), and their functions could be summarized into seven core categories (**Supplementary File 4**, **Supplementary Figure S1**): The first category was pathways related to Culex genetic information transmission and chromosome regulation, involving DNA replication, transcriptional regulation, and chromosome assembly, which might be related to the regulatory mechanism of species-specific gene expression. The second category was signal transduction and stimulus response pathways, including G protein-coupled receptor signaling pathway and chemical stimulus response, which were directly related to the function of perceiving external signals. The third category was metabolic process-related pathways, covering carbohydrate metabolism, lipid metabolism, etc., which provided energy support for physiological activities. The fourth category was enzyme activity and molecular binding pathways, such as hydrolase activity and odorant molecular binding, among which the enrichment of the “odorant molecular binding” pathway further confirmed the effect of species differences on olfactory molecular recognition function. The fifth category was cell structure assembly and movement pathways, involving microtubule assembly and cytoskeleton formation, which might be related to species-specific structural differentiation. The sixth category was transmembrane transport and substance transport pathways, including ion transport and small molecule transmembrane transport, which assisted the transmission of signal molecules inside and outside cells. The seventh category was cell proliferation and regulation pathways, which participated in cell division and growth regulation, providing guarantee for tissue development.

### Composition and diversity analysis of gut microbiome in mosquitoes

Metagenomic sequencing analysis was performed on the gut microbiome of male and female samples from three *Cx.* subspecies (*Cx. quinquefasciatus*, *Cx. p. molestus*, *Cx. p. pallens*), focusing on the core characteristics of community composition and differences in diversity.

**Figure 2:**
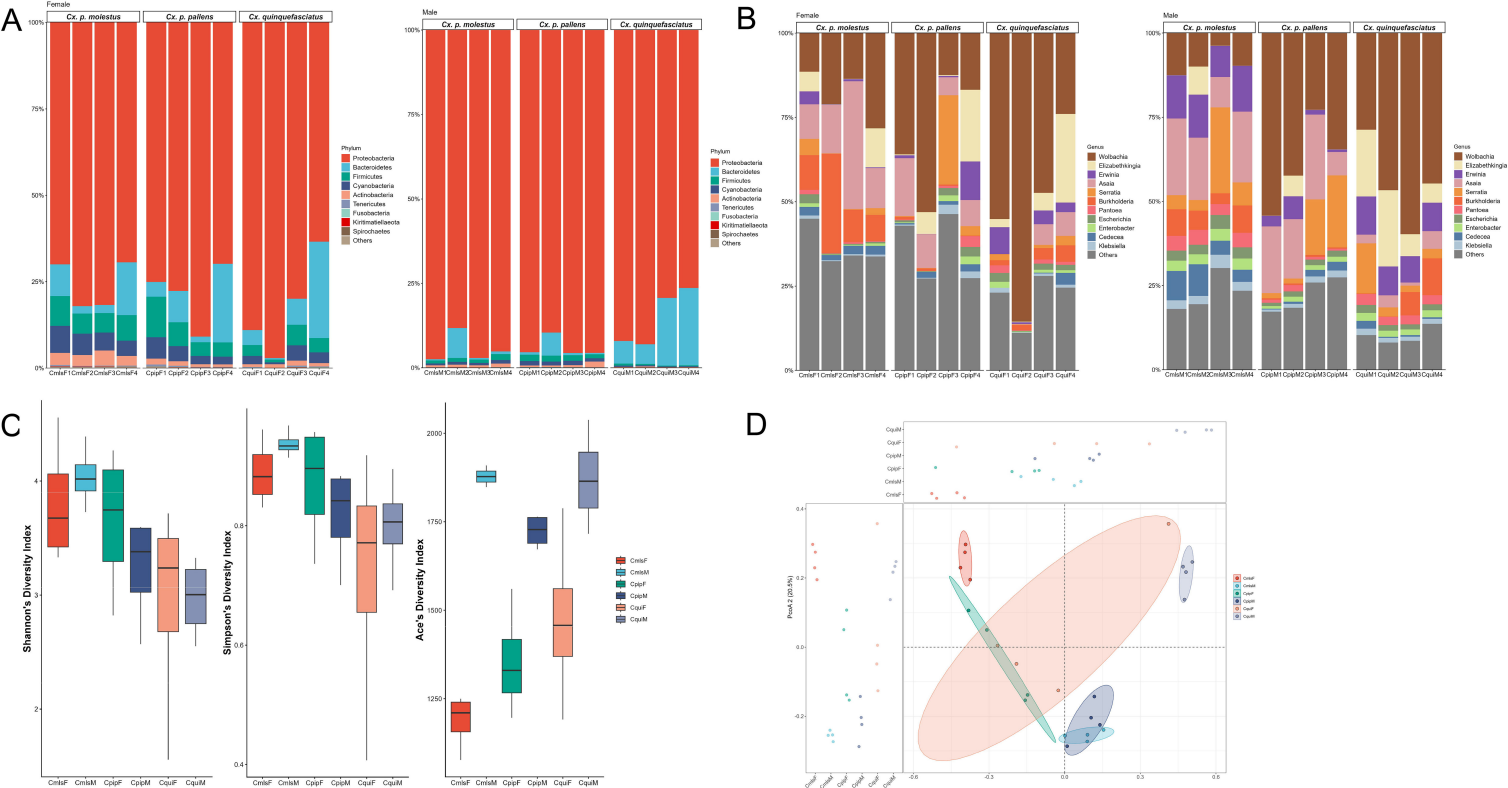
Composition and diversity analysis of gut microbiome in mosquitoes. **(A)** Relative abundance of dominant bacterial phyla in the gut of mosquitoes. **(B)** Relative abundance of dominant bacterial genera in the gut of mosquitoes. **(C)** α-diversity indices of gut microbiome in mosquitoes (from left to right: Shannon index, Simpson index, ACE index). **(D)** PcoA analysis of gut microbial community structure in mosquitoes.

At the phylum level, a total of 29 bacterial phyla were detected in the gut microbiome of the three *Cx.* subspecies. Among them, the top 9 dominant phyla accounted for 99.87% of the total abundance, representing the core components of the gut microbiome community. Ranked by abundance from highest to lowest, these 9 dominant phyla were: Proteobacteria, Bacteroidetes, Firmicutes, Cyanobacteria, Actinobacteria, Tenericutes, Fusobacteria, Kiritimatiellaeota, and Spirochaetes (**Figure 2A**). Further analysis of the effects of species and sex factors on the phylum-level microbiota revealed that although there were slight differences in the relative abundance of these 9 dominant phyla among the three *Cx.* subspecies (the relative abundance of Bacteroidetes in male *Cx. quinquefasciatus* was slightly higher than that in male *Cx. p. pallens* and male *Cx. p. molestus*), the overall composition structure was highly consistent. Moreover, there was no significant difference in the relative abundance of the 9 dominant phyla between male and female individuals of the same species (p>0.05). These results indicated that the composition of dominant phyla was minimally affected by species and sex factors and remained relatively stable across mosquitoes, serving as the conserved core taxa of the *Culex* gut microbiome.

At the genus level, a total of 738 bacterial genera were annotated in the gut microbiome of the three *Cx.* subspecies. Among them, the top 11 dominant genera accounted for 75.16% of the total abundance, functioning as the main functional taxa of the gut microbiome community. Ranked by relative abundance from highest to lowest, these 11 dominant genera were: *Wolbachia*, *Elizabethkingia*, *Erwinia*, *Asaia*, *Serratia*, *Burkholderia*, *Pantoea*, *Escherichia*, *Enterobacter*, *Cedecea*, and *Klebsiella* (**Figure 2B**). The relative abundance of these 11 dominant genera showed obvious species-specific patterns among the three *Cx.* subspecies. For example, the abundance of *Asaia* was the lowest (only 3.19%) in *Cx. quinquefasciatus*, which was significantly lower than its abundance in *Cx. p. pallens* and *Cx. p. molestus*. The abundance of *Wolbachia* was the lowest (13.77%) in *Cx. p. molestus*, but it was still the most abundant genus in the gut of this species, and its abundance in *Cx. quinquefasciatus* and *Cx. p. pallens* were higher than that in *Cx. p. molestus*. Further statistical analysis showed that there was no significant difference in the abundance of the 11 dominant genera between male and female individuals of the same species (p>0.05), suggesting that differences in dominant genera at the genus level were mainly driven by species factors.

α-diversity analysis based on the Shannon index (community diversity), Simpson index (community dominance), and ACE index (community richness) showed the following results: There was no significant difference in the Shannon index and Simpson index among the three *Cx.* subspecies (p>0.05), indicating that the overall diversity and dominance levels of the gut microbiome were similar across the three species. Within the same species, the ACE index of male mosquitoes was significantly higher than that of female mosquitoes: specifically, the ACE index of male *Cx. quinquefasciatus* was significantly higher than that of females. the ACE index of male *Cx. p. pallens* was significantly higher than that of females. and the ACE index of male *Cx. p. molestus* was significantly higher than that of females (**Figure 2C**). Since the ACE index reflects community species richness, these results suggested that within the same *Cx.* subspecies, the species richness of the gut microbiome in males was significantly higher than that in females, and it was inferred that sex factors exerted a specific regulatory effect on the richness of the gut microbiome.

Results of principal coordinate analysis (PcoA) based on Bray-Curtis distance showed an obvious “sex clustering” pattern at the β-diversity level: samples of conspecific mosquitoes (of the same sex) from different species were clustered closer together in the PcoA plot. All male *Culex* mosquitoes (including male individuals of *Cx. quinquefasciatus*, *Cx. p. molestus*, and *Cx. p. pallens*) formed a relatively concentrated cluster, while all female *Culex* mosquitoes (including female individuals of *Cx. quinquefasciatus*, *Cx. p. molestus*, and *Cx. p. pallens*) formed another independent cluster. In contrast, male and female samples of the same species showed a clear separation trend (**Figure 2D**). Further ANOSIM analysis verified that the effect of sex factors on the gut microbiome community structure was statistically significant (R = 0.62, p<0.01), and the degree of this effect was higher than that of species factors (R = 0.45, p<0.05). It was thus inferred that sex was the key factor driving the differentiation of gut microbiome community structure among the three *Cx.* subspecies, and the gut microbiome community composition of mosquitoes of the same sex was more similar.

### Correlation analysis between antennal transcriptome and gut microbiome

To systematically explore the interaction between the antennal transcriptome and gut microbiome of *Culex* mosquitoes, we first integrated the DEGs among male and female individuals of three *Cx.* subspecies. After removing redundant and low-expression genes, a core gene set containing 3,833 differentially expressed genes was finally constructed.

Meanwhile, we performed differential abundance screening on the gut microbiome data. After excluding low-abundance genera with an abundance of less than 0.01%, a microbial abundance dataset including 366 bacterial genera was established. This dataset covered the 11 dominant genera identified in the previous section (*Wolbachia*, *Elizabethkingia*, *Asaia*, etc.) and 355 medium-to-low abundance genera, comprehensively representing the species composition of the *Culex* gut microbiome.

**Figure 3:**
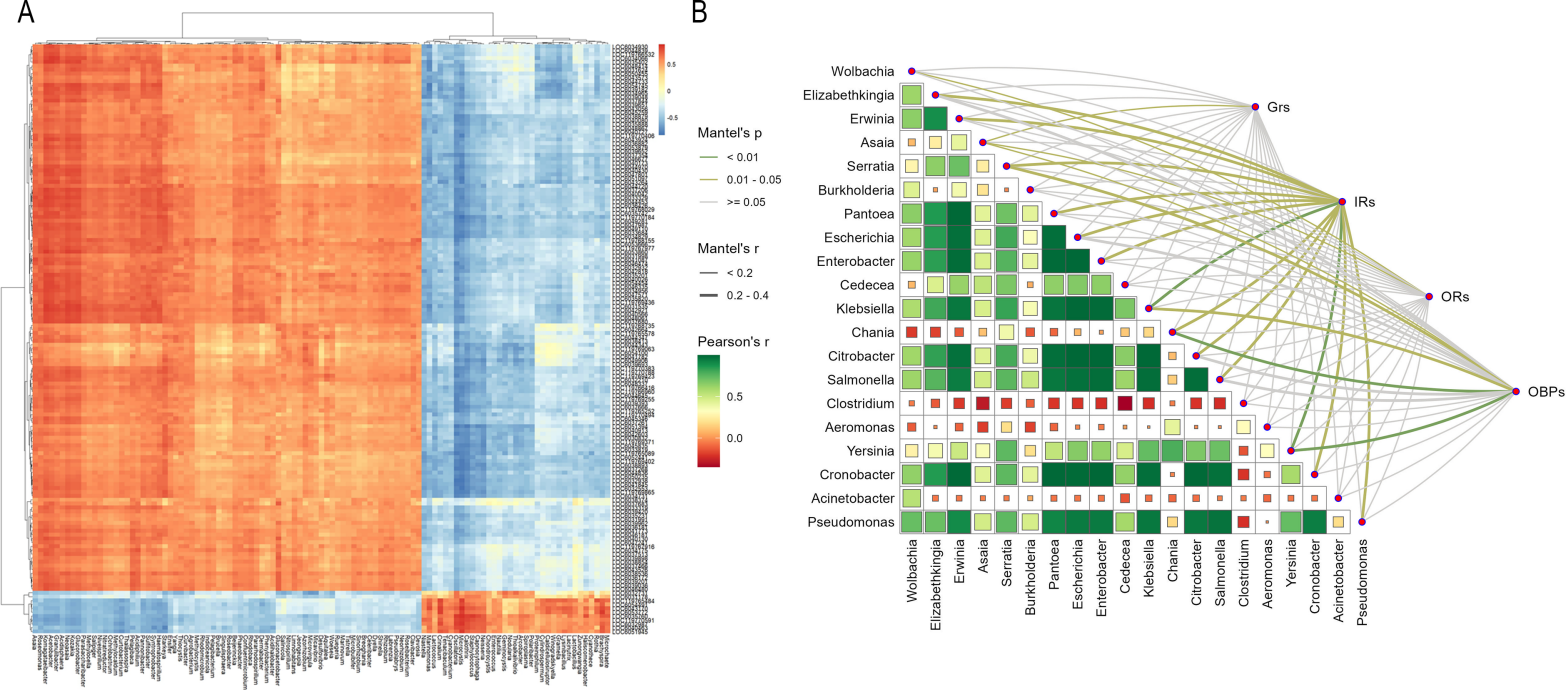
Correlation between gut microbial abundance and antennal gene expression levels. **(A)** The heatmap displays genes and microbes with correlated expression levels and abundances (152 DEGs and 107 bacterial genera were screened out from 3833 DEGs and 366 bacterial genera). Red indicates the degree of positive correlation, and blue indicates the degree of negative correlation. **(B)** The Mantel correlation heatmap shows the association between the core gut bacterial communities of mosquitoes and olfactory related differentially expressed genes. The heatmap presents the correlations among dominant bacterial genera; the color of the lines represents the *p*-value of the correlation between microbes and this category of olfactory genes, and the thickness of the lines represents the *r*-value of the correlation between microbes and this category of olfactory genes.

Based on the aforementioned gene set and microbial dataset, Spearman correlation analysis (screening criteria: |R|>0.6, p<0.05) was conducted to investigate the correlation between them. The results showed that a total of 107 microbial genera and 152 differentially expressed genes exhibited strong correlation relationships, forming 216 significantly correlated “microbe-gene” pairs (**Figure 3A**, **Supplementary File 6**, **Supplementary Table S5**).

### Targeted correlation analysis between olfaction-related genes and core microbes

To precisely explore the impact of the gut microbiome on the olfactory physiological functions of mosquitoes, we further screened 176 olfaction-related genes from the core gene set. These genes included 55 odorant binding protein (OBP) genes, 66 odorant receptor (OR) genes, 18 ionotropic receptor (IR) genes, and 37 gustatory receptor (GR) genes—all of which are key functional genes regulating the recognition and transmission of mosquito olfactory signals (**Supplementary File 5**, **Supplementary Table S4**).

At the microbial level, the top 20 genera with the highest abundance in the gut microbiome were selected for targeted correlation analysis. The cumulative abundance of these core microbes accounted for 90.37% of the total abundance of the gut microbiome, which could represent the main functional taxa of the gut microbial community (including dominant genera such as *Wolbachia*, *Elizabethkingia*, *Erwinia*, and *Asaia*).

A further detailed analysis of the correlation between different types of olfaction-related genes and core microbial genera (screening criteria: |R|>0.6, p<0.05) revealed that various gene types showed significant correlations with specific microbial genera: OR genes: Significantly correlated with *Wolbachia* and *Asaia*. GR genes: Significantly positively correlated only with *Asaia*. OBP genes: Significantly correlated with four microbial genera, namely *Asaia*, *Serratia*, *Chania*, and *Yersinia*. IR genes: Had the widest range of correlated microbial genera, showing significant correlations with 16 genera in total, including *Wolbachia*, *Elizabethkingia*, *Erwinia*, *Serratia*, *Burkholderia*, *Pantoea*, *Escherichia*, *Enterobacter*, *Cedecea*, *Klebsiella*, *Chania*, *Citrobacter*, *Salmonella*, *Yersinia*, *Cronobacter*, and *Pseudomonas* (**Figure 3B**).

## Discussion

By integrating antennal transcriptome sequencing and gut metagenomic data, this study systematically analyzed the expression characteristics of olfaction-related genes, differences in gut microbial communities, and their intrinsic associations in male and female individuals of three major urban vector mosquito species, providing a new perspective for uncovering the associations between gut microbiota and olfactory function-related genes in mosquitoes.

Transcriptomic analysis revealed that all three *Cx.* species exhibited significant sexually dimorphic DEGs, with *Cx. quinquefasciatus* showing the highest number of DEGs between males and females. This observation may be associated with the species’ stronger environmental adaptability and reproductive capacity. Notably, differences in blood-feeding behavior among the three *Cx.* species were primarily concentrated in females—female mosquitoes of different species displayed distinct divergence in host preference, blood-feeding frequency, and post-feeding physiological metabolism regulation (Soghigian et al., 2023), while males exclusively fed on plant sap with minimal variation in behavioral habits (Reeves and Burkett-Cadena, 2024; Shannon et al., 2024). This physiological and behavioral dimorphism was directly reflected at the gene expression level: the number of DEGs between females of the three *Cx.* species was significantly higher than that between males. These results suggest that females have evolved more extensive gene expression variations to adapt to blood-feeding-related specific physiological needs, such as host recognition, blood digestion, and ovarian development (Stephenson et al., 2019; Muturi et al., 2021), while males exhibit relatively limited gene expression differences due to their more conserved ecological niche and behavioral patterns (Wahid et al., 2003; Vaníčková et al., 2017).

The 345 cross-species shared sex-related DEGs showed consistent expression trends. Genes highly expressed in females were likely involved in female-specific physiological processes, including ovarian development and blood-feeding behavior regulation (Vogel et al., 2015; Wei et al., 2024), while genes highly expressed in males were closely associated with olfaction-related functions in courtship and mating (Klowden, 1999). These findings indicate that these genes serve as core regulatory factors maintaining sexual dimorphism in *Cx.* mosquitoes.

GO and KEGG enrichment analyses demonstrated that the shared DEGs were significantly enriched in pathways related to odor binding, signal transduction, and xenobiotic metabolism. Particularly, the enrichment of olfactory-related molecular functions confirmed the antenna’s role as the core olfactory organ (Konopka et al., 2023). It is important to note that while blood-feeding provides females with nutrients essential for ovarian development, it also exposes them to foreign factors in host blood and residual xenobiotics in the environment (Wu et al., 2019). Consequently, xenobiotic metabolism and degradation have become critical functions for females to maintain physiological homeostasis, which is further supported by the significant enrichment of DEGs in KEGG pathways such as “Drug Metabolism” and “Xenobiotic Metabolism” (Goldman et al., 2025). This further illustrates that females enhance the expression of xenobiotic metabolism-related genes to cope with potential physiological risks associated with blood-feeding. Interspecific DEG analysis showed that the transcriptomic clustering distance between *Cx. p. pallens* and *Cx. p. molestus* was closer, consistent with their relatively close taxonomic relationship (subspecies of *Cx. pipiens*) (Soghigian et al., 2023). For *Cx. quinquefasciatus*, a tropical and subtropical species, the specifically highly expressed Module 1 genes may underlie its adaptation to warm and humid environments (Gorris et al., 2021). Additionally, the “species-sex” clustering pattern indicated that antennal gene expression in *Cx.* mosquitoes are co-regulated by species-specific factors and sexual differentiation, which may serve as an important molecular basis for interspecific differences in ecological niche differentiation and behavioral habits.

Metagenomic analysis revealed the core compositional characteristics of the gut microbiome in the three *Cx.* species: at the phylum level, Proteobacteria, Bacteroidetes, and Firmicutes constituted the dominant flora, accounting for a total of 99.87% of the microbial abundance. These phyla were minimally affected by species and sex factors, reflecting the conservation of the gut microbial community in *Cx.* mosquitoes. This composition is consistent with the general pattern of insect gut microbiomes, where Proteobacteria dominate (Wang et al., 2011; Wu et al., 2019b; Zhao et al., 2022; Mandal and Schmidt, 2023). These microbial groups typically participate in basic host physiological processes such as nutrient metabolism and immune defense, and their stability is crucial for mosquito survival (Harrison et al., 2023).

At the genus level, the top 11 dominant genera among 738 annotated genera accounted for 75.16% of the total abundance, showing distinct species-specific patterns: *Asaia* had the lowest abundance (3.19%) in *Cx. quinquefasciatus*, while *Wolbachia* had the lowest abundance (13.77%) in *Cx. p. molestus* but remained a dominant genus. Importantly, previous studies have confirmed that *Asaia* in the gut microbiome of most insects harbors the pyrethroid hydrolase gene, which encodes a hydrolase capable of degrading pyrethroid insecticides and conferring insecticide resistance to the host (Comandatore et al., 2021). In this study, *Asaia* served as a core dominant genus in all three *Cx.* species, and it is hypothesized that *Asaia* may enhance the resistance of *Cx.* mosquitoes to commonly used pyrethroid insecticides through the expression of pyrethroid hydrolase. The significantly lower abundance of *Asaia* in *Cx. quinquefasciatus* (a tropical and subtropical species) compared to *Cx. p. pallens* and *Cx. p. molestus* (temperate species) may be attributed to differences in insecticide application strategies across climatic zones (Liu, 2015; Helps et al., 2020; Barathi et al., 2024). In subtropical regions, where mosquitoes are active year-round, a wider variety of insecticides are used with higher rotation frequencies (Abdulai et al., 2023), potentially reducing reliance on *Asaia*-mediated pyrethroid resistance and thereby decreasing the selective advantage of *Asaia* in the gut of *Cx. quinquefasciatus*.

Furthermore, *Wolbachia* not only acted as a core dominant genus in the three *Cx.* species studied but also was frequently identified as a key dominant group in gut microbiome analyses of other mosquito species such as *Aedes aegypti* and *Aedes albopictus* (Ahmad et al., 2017; Mancini et al., 2020; Zhang et al., 2022; Pascar et al., 2023). As a gram-negative endosymbiotic bacterium widely present in mosquitoes, numerous studies have confirmed that *Wolbachia* plays a critical role in regulating mosquito physiological functions—for instance, it induces cytoplasmic incompatibility (CI) to affect mosquito reproductive efficiency and enhances host resistance to pathogens such as dengue virus and Zika virus (Moreira et al., 2009; Ant et al., 2020; Leitner et al., 2021; Sicard et al., 2021; Fox et al., 2024; Mejías et al., 2025). Notably, *Cx. p. molestus* exhibits a unique autogenous trait (Sawabe and Moribayashi, 2000; Haba et al., 2025), while *Wolbachia*-mediated cytoplasmic incompatibility primarily functions by regulating post-mating gamete compatibility in the host. For autogenous species, the reliance on *Wolbachia*-mediated “mating compatibility regulation” during reproduction is significantly reduced, which may weaken the selective pressure on *Wolbachia* within the host and consequently lead to a lower abundance of *Wolbachia* compared to anautogenous species. The lowest abundance of *Wolbachia* in *Cx. p. molestus* observed in this study is hypothesized to be closely associated with the subspecies’ autogenous trait, reflecting the coevolutionary relationship between microbial abundance and host reproductive strategies. Meanwhile, interspecific differences in *Wolbachia* abundance may further impact the reproductive efficiency, stress resistance, and disease transmission potential of different *Cx.* species. No significant differences in the abundance of dominant gut genera were observed between males and females of the same species, indicating that the species-specificity of the *Cx.* gut microbiome is primarily determined by genetic background and ecological environment rather than sex-related physiological differences.

Diversity analysis showed no significant differences in the Shannon and Simpson indices of gut microbes among the three *Cx.* species. However, males of the same species exhibited a significantly higher ACE index than females, suggesting greater species richness of gut microbes in male *Cx.* mosquitoes. This may be related to differences in nutritional requirements between males and females: females require blood-feeding to obtain proteins, leading to the specialization of gut microbial communities toward blood digestion (Dadd, 1980; Klowden, 1993; van Schoor et al., 2020; Romoli et al., 2024). In contrast, males only feed on plant sap, and a more diverse microbial community facilitates the metabolism of various carbohydrates (Baik et al., 2024; Pullmann-Lindsley et al., 2024). The “sex-clustering” pattern in β-diversity is particularly noteworthy—sex exerts a stronger influence on community structure than species identity. This finding differs from the species-dominated differentiation pattern reported in previous insect microbiome studies (Medeiros et al., 2023; Magura et al., 2024), implying potential sex-related functional specialization of the gut microbiome in *Cx.* mosquitoes and providing new clues for investigating microbe-host sexual interactions.

Multi-omics association analysis constructed an interaction network involving 3,833 DEGs and 366 bacterial genera, identifying 107 microbial genera significantly associated with 152 genes. This indicates a close association between the gut microbiome and antennal gene expression in *Cx.* mosquitoes, which are consistent with previous research findings (Das De et al., 2018; Sharma et al., 2020; Das De et al., 2022). Gut microbes can influence the host not only through local metabolism but also by producing short-chain fatty acids, neurotransmitter precursors, or immune signaling molecules, which are transported via humoral circulation—providing potential molecular links underlying the observed associations.

Specifically, ORs as core components of olfactory signal recognition, possess a unique “complex receptor function”, a single olfactory neuron can simultaneously recognize multiple volatile substances (Herre et al., 2022; Zhao J. et al., 2024). This trait enables mosquitoes to detect mixed characteristic odors released by hosts from a long distance and quickly locate targets in complex environments (Pelletier et al., 2010; Maguire et al., 2022). Additionally, it provides “functional redundancy protection”, even if some ORs are impaired by environmental stress, other related ORs can still maintain core recognition functions (Herre et al., 2022). In this study, ORs were primarily associated with *Wolbachia* and *Asaia*. It is hypothesized that the abundance of these two core genera is associated with the expression of OR-controlling transcription factors, with this association potentially reflecting a correlative pattern between microbial metabolites and OR complex receptor function-related gene expression—consistent with the observed strong Spearman correlation between *Wolbachia/Asaia* and OR genes.

GR was also found to be associated with *Wolbachia* and *Asaia* in this study. The 37 annotated GRs in this study covered 8 types, including GR2, GR22, GR24, and GR28. These GRs mediate the acceptance or avoidance behavior of mosquitoes toward host secretions and plant sap by recognizing different olfactory molecules (Jones et al., 2007). Among them, GR28 exerts non-gustatory functions in the nervous system, participating in proprioception, hygroreception, and sensory signal integration (Thorne and Amrein, 2008). GR66a acts as a bitter taste receptor to help mosquitoes avoid toxic substances (Apostolopoulou et al., 2015). GR43 and GR64 recognize sweet tastes for nutrient acquisition (Jiao et al., 2007). GR39a regulates male courtship behavior by receiving female-released stimulatory inhibitor pheromones (Watanabe et al., 2011). It is hypothesized that gut microbial metabolites can activate specific pathways, regulate the excitability of GR-associated neurons and thereby influencing the efficiency of signal transduction, enabling mosquitoes to respond more accurately to gustatory signals in the environment.

As “molecular carriers” for olfactory signal transduction, the expression levels of OBPs were found to be associated with the abundance of four genera in this study: *Asaia, Serratia, Chania*, and *Yersinia*. Their functions extend beyond simple odor molecule binding and transport. The OBP lush annotated in this study has been confirmed in Aedes aegypti research to be relevant to normal female fecundity and fertility, and it may also be involved in modulating blood-feeding behavior and potentially facilitating the shedding of dengue virus type 2 and Zika virus particles into mosquito saliva (Sim et al., 2012; Dong et al., 2021). Combining the functional characteristics of Serratia with the observed associations in this study, it is hypothesized that *Serratia marcescens* (a species within the *Serratia* genus) may be linked to mosquito physiological behavior and disease transmission potential through a dual hypothetical scenario: on one hand, the bacterial functional factor SmEnhancin secreted by S. marcescens has been reported to degrade the mucin layer on the surface of mosquito gut cells, which could disrupt the integrity of the gut barrier and thereby potentially favor flavivirus infection (Wu et al., 2019a). This may form a potential “infection-transmission” synergistic relationship with the function of OBP lush in facilitating viral particle shedding. On the other hand, Serratia may produce lipid signaling molecules through metabolism, which could be transported via the circulatory system to pathways related to the brain’s reproductive and olfactory integration, potentially correlating with the transcription and translation of OBP lush while being associated with the brain’s regulatory signals for blood-feeding behavior (Kozlova et al., 2021). This proposed dual role of “potential olfactory association-viral facilitation” suggests that *Serratia* could be a noteworthy genus linking mosquito host localization ability and disease transmission capacity, which merits further investigation.

The expression level of IRs is widely correlated with the abundance of 16 bacterial genera. Among the 18 annotated IRs, members of the IR75a family accounted for a high proportion (14/18). Previous studies in Drosophila melanogaster have confirmed that IR75a is a specific receptor for short-chain fatty acid (SCFA)-type odor molecules such as acetic acid and propionic acid and can serve as a key component of the olfactory receptor complex to participate in environmental odor recognition (Abuin et al., 2011; Prieto-Godino et al., 2016). Combined with the association results of this study, it is hypothesized that gut microbes may be linked to the expression of IR75a in the antennae of Culex mosquitoes through the metabolic production of short-chain fatty acids or may interact with signal molecules to be associated with the olfactory signaling pathway mediated by IR75a. This could potentially affect the mosquitoes’ ability to perceive host secretions and oviposition site environments, providing specific molecular clues for the observed associations.

Overall, the extensive associations between olfaction-related genes (particularly IR75a, ORs, OBPs such as OBP lush, and GRs such as GR28 and GR39a) and microbial genera collectively point to a complex network of relationships underlying mosquito olfactory function. *Wolbachia*, as a core genus associated with both ORs and IRs, may be involved in the brain’s signal integration related to mating recognition or host localization. *Asaia*, with its multiple associations with OBPs, ORs, and GRs, could potentially play a role in the entire chain of olfactory signal processing from “carrier binding-receptor activation-brain decision-making”. *Serratia*, through the proposed dual pathway of “SmEnhancin-mediated viral facilitation and OBP-associated olfactory-related processes”, may act as a key hub linking mosquito environmental adaptation and disease transmission. These results highlight the broad associations between gut microbes and antennal olfaction-related gene expression, as well as the potential link between microbial presence and local pathogen infection, laying a foundation for further exploration of the underlying biological relationships.

Mantel analysis further showed that the core gut microbial community was significantly correlated with the expression of olfaction-related genes, and this correlation was stronger in female *Cx.* mosquitoes than in males. This aligns with the biological trait that females depend on olfaction to complete key reproductive behaviors such as blood-feeding and oviposition, reflecting a more pronounced associative relationship between gut microbes and olfactory gene expression in females—likely linked to their olfactory-dependent physiological demands. This sex-specific association may represent a potential biological link relevant to environmental adaptability and reproductive success in female *Cx.* mosquitoes.

Moreover, this sex-specific pattern provides targeted directions for mosquito control: for example, regulating the abundance of Asaia and Wolbachia to interfere with potential molecular links involving short-chain fatty acids or signaling molecules, which could affect female host localization ability, reproductive efficiency, or male courtship behavior. Meanwhile, targeted intervention measures for *Serratia* (e.g., inhibiting SmEnhancin secretion or activity) may block its reported facilitation of flavivirus infection, thereby reducing mosquito population density and disease transmission risk through multiple aspects including reproduction, feeding, and viral transmission.

In this study, non-blood-fed individuals of *Cx. quinquefasciatus, Cx. p. pallens*, and *Cx. p. molestus* were selected as research objects. Through integrated analysis of antennal transcriptomics and gut metagenomics, we identified extensive associations between gut microbes and mosquito olfactory gene expression—associations that may be relevant to the differences in hematophagous capacity among different mosquito taxa. It is important to note that the current findings are limited to non-blood-fed mosquitoes, as blood-feeding is known to reshape gut microbial composition and olfactory-related physiological processes. In future studies, we will expand the research system to include blood-fed mosquito samples, aiming to comprehensively explore how blood ingestion modulates the associations between gut microbiota and olfactory gene expression, and further clarify the potential biological relevance of these dynamic interactions in the context of mosquito hematophagy and pathogen transmission. These current findings provide key clues for initiating investigations into “microbe-olfaction” interactions in mosquitoes, laying a foundation for subsequent in-depth exploration of the underlying biological relationships.

## Data Availability

The datasets presented in this study can be found in online repositories. The names of the repository/repositories and accession number(s) can be found below: https://ngdc.cncb.ac.cn/bioproject/, PRJCA050988.

## Supplementary material

Supplementary Figure 1 GO Enrichment Analysis for Species-Specific DEGs. **(A)** Female *Cx. p. pallens vs* female *Cx. p. molestus*. **(B)** Female *Cx. p. pallens vs* female *Cx. quinquefasciatus*. **(C)** Female *Cx. quinquefasciatus vs* female *Cx. p. molestus.***(D)** Male *Cx. p. pallens vs* male *Cx. p. molestus*. **(E)** Male *Cx. p. pallens vs* male *Cx. quinquefasciatus*. **(F)** Male *Cx. quinquefasciatus vs* female *Cx. p. molestus.*
