# Supplementary figures and images for: Multi-omics profiling reveals associations between gut microbiota and olfactory gene expression in mosquitoes
Source: Front Cell Infect Microbiol. 2026 Jan 26;15:1745848. doi: 10.3389/fcimb.2025.1745848 (PMC12883841; doi:10.3389/fcimb.2025.1745848)

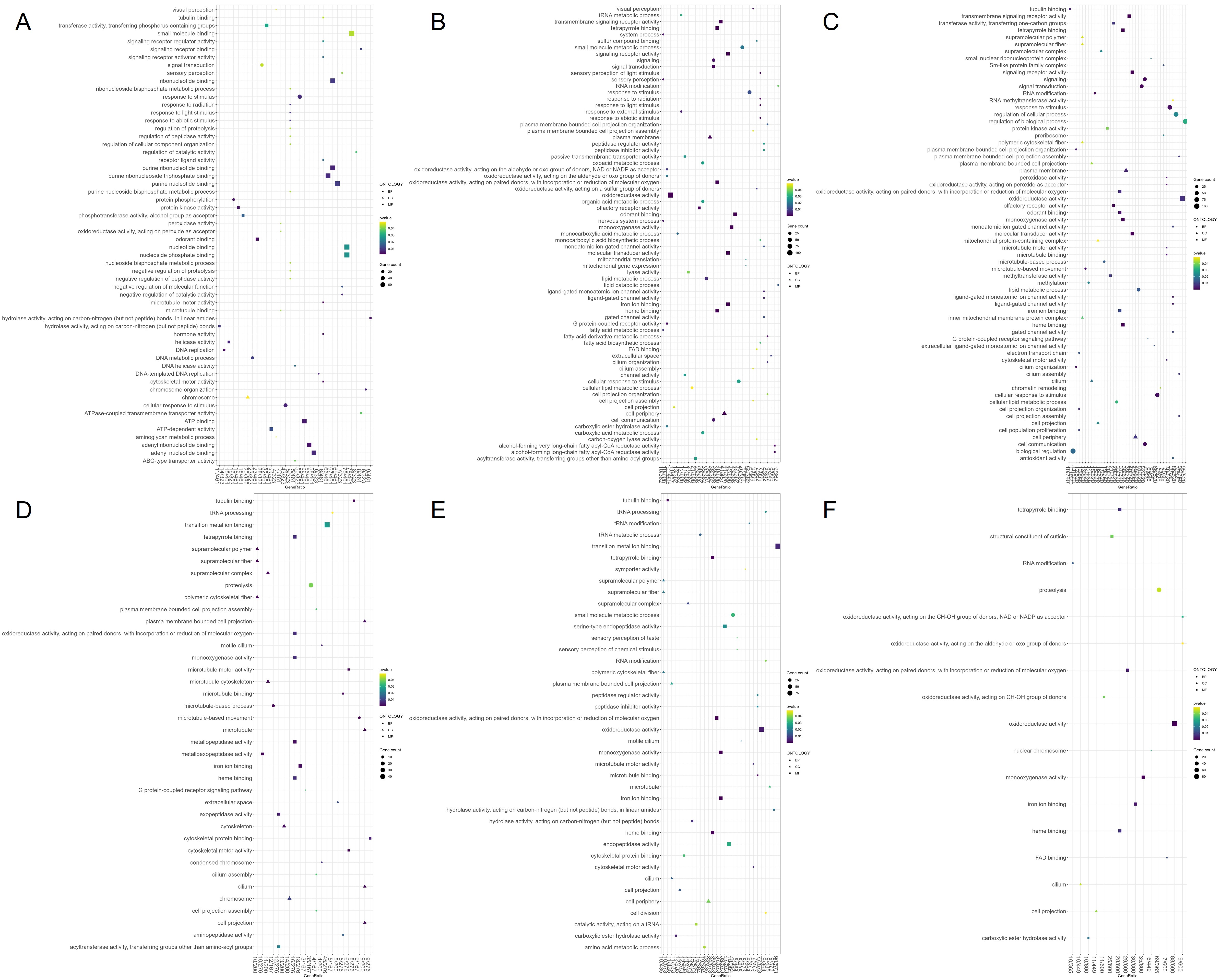

Supplement: Supplementary Figure 1 — GO Enrichment Analysis for Species-Specific DEGs. (A) Female Cx. p. pallens vs female Cx. p. molestus. (B) Female Cx. p. pallens vs female Cx. quinquefasciatus. (C) Female Cx. quinquefasciatus vs female Cx. p. molestus.(D) Male Cx. p. pallens vs male Cx. p. molestus. (E) Male Cx. p. pallens vs male Cx. quinquefasciatus. (F) Male Cx. quinquefasciatus vs female Cx. p. molestus. [file Image1.jpeg]
